# Association Between Iron-Related Protein Lipocalin 2 and Cognitive Impairment in Cerebrospinal Fluid and Serum

**DOI:** 10.3389/fnagi.2021.663837

**Published:** 2021-06-25

**Authors:** Sofia Pereira das Neves, Ricardo Taipa, Fernanda Marques, Patrício Soares Costa, Joel Monárrez-Espino, Joana A. Palha, Miia Kivipelto

**Affiliations:** ^1^Life and Health Sciences Research Institute (ICVS), School of Medicine, University of Minho, Braga, Portugal; ^2^ICVS/3B’s—PT Government Associate Laboratory, Braga/Guimarães, Portugal; ^3^Department of Neurosciences, Centro Hospitalar do Porto, Porto, Portugal; ^4^Department of Health Research, Christus Muguerza Hospital—University of Monterrey, Chihuahua, Mexico; ^5^Division of Clinical Geriatrics, Center for Alzheimer Research, Department of Neurobiology, Care Sciences and Society, Karolinska Institutet, Stockholm, Sweden; ^6^Theme Aging, Karolinska University Hospital, Stockholm, Sweden

**Keywords:** Alzheimer’s disease, mild-cognitive impairment, cognition, lipocalin 2, biomarker

## Abstract

A worldwide increase in longevity is bringing novel challenges to public health and health care professionals. Cognitive impairment in the elderly may compromise living conditions and precede Alzheimer’s disease (AD), the most prevalent form of dementia. Therefore, finding molecular markers associated with cognitive impairment is of crucial importance. Lipocalin 2 (LCN2), an iron-related protein, has been suggested as a potential marker for mild cognitive impairment (MCI) and AD. This study aimed at investigating the association between LCN2 measured in serum and cerebrospinal fluid (CSF) with cognitive impairment. A cross-sectional design based on two aging cohorts was used: individuals diagnosed with subjective cognitive complaints (SCC), MCI, and AD from a Swedish memory clinic-based cohort, and individuals diagnosed with SCC and AD from a Portuguese cohort. Binary logistic [for the outcome cognitive impairment (MCI + AD) in the Swedish cohort and AD in the Portuguese cohort] and multinomial logistic (for the outcomes MCI and AD) regression analyses were used. No associations were found in both cohorts when controlling for sex, education, and age. This explanatory study suggests that the association between serum and CSF LCN2 concentrations with cognitive impairment reported in the literature must be further analyzed for confounders.

## Introduction

The increase in worldwide longevity attained during the last 100 years has been a major public health achievement (World Health Organization, 2011; Prince et al., 2015). Unfortunately, aging is associated with cognitive decline (Harada et al., 2013). Yet, cognitive trajectories throughout aging are not constant within or between individuals; these seem to depend on factors whose interactions remain unknown. While some are thought to arise from genetic factors (Pilling et al., 2012), others depend on the individual’s interaction with the environment (Kulmala et al., 2019).

Impaired cognition may compromise the quality of life of individuals (León-Salas et al., 2015), and can ultimately evolve to Alzheimer’s disease (AD; Rabin et al., 2017), which is among the most prevalent diseases of the elderly population (World Health Organization, 2020). Of relevance, mild cognitive impairment (MCI), a condition characterized by cognitive decline but in which individuals maintain their normal lives, is even more common than AD (Katz et al., 2012). In about half of the cases, MCI progresses to AD (Gauthier et al., 2006). Identifying molecular biomarkers associated with MCI and early AD that can be used as early indicators of AD disease progression is essential to carry out timely interventions.

In addition to the hallmarks of AD (i.e., amyloid beta peptide and tau protein), several other molecules have the potential to become useful biomarkers, including lipocalin 2 (LCN2), an acute-phase protein involved in iron homeostasis (Mesquita et al., 2012, 2014; Ferreira et al., 2013). Animal models have shown that changes in LCN2 concentrations in the brain correlate well with behavioral changes, such as anxiety (Mucha et al., 2011; Ferreira et al., 2013) and memory (Choi et al., 2011; Naudé et al., 2012; Ferreira et al., 2013). LCN2 has also been implicated in various molecular mechanisms relevant to the pathophysiology of AD (Lee et al., 2009, 2011, 2012; Rathore et al., 2011; Roudkenar et al., 2011; Bi et al., 2013), namely LCN2 can amplify neuroinflammation (Lee et al., 2011), participate in reactive astrocytosis (Lee et al., 2009; Bi et al., 2013), promote neuronal cell death (Bi et al., 2013), and mediate amyloid beta (Aβ) toxicity (Mesquita et al., 2014). LCN2 concentrations were also increased in post-mortem brain tissue of AD patients, in regions associated with brain pathology such as the hippocampus (Naudé et al., 2012). In fact, serum LCN2 concentrations were increased in patients with MCI, but not with AD, when compared with cognitively healthy individuals (Choi et al., 2011). Additionally, LCN2 plasma levels were increased in preclinical AD, classified accordingly to the CSF Aβ_42_, total tau and phosphorylated tau levels, compared to controls (Eruysal et al., 2019). However, clinical evidence is still contentious, as this finding was not replicated by a later study (Naudé et al., 2012) whereby no changes in serum LCN2 were seen, but instead, a decrease in cerebrospinal fluid (CSF) concentrations among individuals with MCI and AD was recorded. It is in this context that the present study aims at clarifying the relationship between LCN2 levels, measured in serum and CSF, and cognitive performance in MCI and AD.

## Subjects, Materials and Methods

### Study Design and Study Population

A design based on two aging cohorts was used. For the Swedish cohort, a cross-sectional sample of consecutive patients with available samples was obtained from the Memory Clinic (Karolinska University Hospital, Huddinge, Stockholm) using GEDOC, a database, and biobank for geriatric research (Almkvist and Tallberg, 2009). GEDOC is an electronic database that has existed at the Theme Aging (previous geriatric clinic) at Karolinska University Hospital since the 90s and includes patients who have visited the memory clinic for examinations and given their informed consent. Individuals evaluated within the period 2005–2015 who fulfilled the inclusion criteria were eligible; this sample was representative of the Memory Clinic population. For the Portuguese cohort, patients were prospectively enrolled from the dementia outpatient clinic of the Department of Neurology at Centro Hospitalar Universitário do Porto (CHUP) between February 2013 and April 2017.

*Inclusion criteria*: Individuals with a clinical diagnosis of subjective cognitive complaints (SCC), MCI, or AD with available serum and CSF samples for biochemical analyses. Complete data on neuropsychological performance, age, sex, and the number of years of formal education, were also requirements for inclusion.

**Figure 1:**
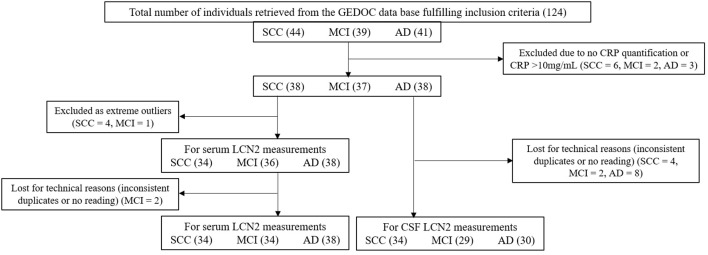
Flow chart of the Swedish study participants.

**Figure 2:**
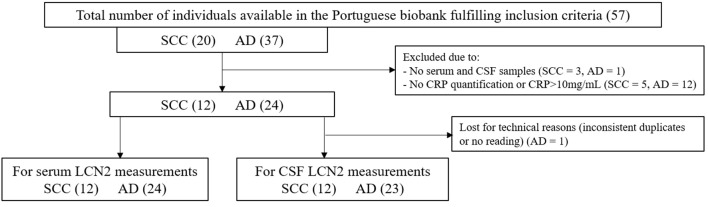
Flow chart of the Portuguese study participants.

*Exclusion criteria*: Individuals with serum C-reactive protein (CRP) concentrations above 10 mg/ml, as this is a sign of inflammatory response that can interfere with the analysis of LCN2, an acute-phase protein (US Food and Drug Administration, 2005). The overall number of samples available for analyses can be seen in Figure 1 for the Swedish cohort and in Figure 2 for the Portuguese cohort.

### Neuropsychological Assessment and Diagnostic Criteria

All participants underwent a clinical investigation that included medical history, neuropsychological testing, physical and neurological evaluation, blood collection, and lumbar puncture to obtain CSF. Diagnoses were established by multidisciplinary teams of medical doctors, clinical neuropsychologists, speech therapists, and specialized nurses.

Swedish participants met the criteria for dementia subtype AD, as stated in the Diagnostic and Statistical Manual of Mental Disorders version IV (DSM IV; American Psychiatric Association, 2005). For Portuguese participants, the diagnosis of AD was established using the National Institute on Aging (NIA)-Alzheimer’s Association 2011 criteria (McKhann et al., 2011), including a duration of symptoms ≤4 years. Clinical criteria used were identical in both groups, but additional biomarkers of disease were available for the Portuguese subjects.

Individuals with MCI, who did not fulfill the DSM IV criteria for AD, reported (themselves or through a proxy) current impairment and declining ability to perform objective cognitive tasks, or minimal impairment in complex instrumental functions that do not compromise basic daily living activities (Petersen, 2004; Petersen et al., 2014).

Participants who reported SCC, but who did not show objective impairment on cognitive tests or met AD and MCI criteria, were considered here as the reference group. All participants were characterized according to clinical criteria, neuropsychological testing and underwent CSF AD biomarkers study, which were all normal. Furthermore, these subjects had a follow-up period of at least 24 months to ensure the absence of any pathological neurological diagnosis.

### Sample Size Calculations

For the Swedish cohort, assuming a conservative perspective of a Cohen’s *f* = 0.5, a minimum of 22 samples per group would be needed. Considering the LCN2 values reported in the literature, ANOVA comparison, alpha = beta = 0.05, Cohen’s *f* of 0.786 for serum and 1.795 for CSF, a minimum of 10 samples per group would be required for serum and of three samples per group for CSF (SCC, MCI and AD groups).

For the Portuguese cohort, assuming the same conservative perspective of a Cohen’s *d* = 1 (Cohen’s *f* = 0.5), a minimum of 23 samples per group would be needed. Considering LCN2 values reported in the literature, alpha = beta = 0.05, Cohen’s *d* of 2.27 (Cohen’s *f* = 1.135) for serum and 3.36 (Cohen’s *f* = 1.680) for CSF a minimum *n* = 6 per group for serum and *n* = 3 por CSF (SCC and AD groups) would be needed.

### Biological Fluid Collection and Biochemical Measurements

Venous blood and lumbar CSF were collected in the morning on the same day, or within the same week of the neuropsychological assessment, under usual conditions (non-fasted state) following standard procedures. Blood was primarily collected for clinical evaluation during the diagnostic process; it was centrifuged at 3,000 rotations per minute for 15 min (min) at room temperature (RT) to separate the serum. Both serum and CSF samples were kept at −75°C until analyses.

LCN2 was measured in serum and CSF using an enzyme-linked immunosorbent assay (ELISA) in duplicate samples. The primary antibody (1:200 in PBS; MAB17571, R&D Systems, Minneapolis, MN, USA) was incubated overnight at RT in 96 well plates. The blocking was performed with 1% BSA in PBS for 2 h (h) at RT. CSF samples (1:2) and standards (Recombinant Human LCN2/NGAL, 1757-LC, R&D Systems) were analyzed in duplicates and were diluted in blocking solution and incubated for 2 h at RT. The secondary antibody (1:700 in blocking solution; BAF1757, R&D Systems) was incubated for 2 h at RT. Streptavidin-peroxidase (1:2,000 in PBS; S2438, Sigma-Aldrich) was incubated for 30 min in the dark. All steps were followed by a washing step with PBS-Tween 20 0.05%. The 3,3′,5,5′-tetramethylbenzidine (Sigma Aldrich) substrate was incubated for 30 min in the dark. The reaction was stopped by adding sulfuric acid (2 M), and the absorbance was read in a multiplate reader at 450 nm, using the 570 nm as reference. The average variation of optical density between duplicates of the same sample was below 5%. In one ELISA plate there was an abnormal reading of the outer row that originated inconsistent duplicates; these samples were not included in the analyses (Figure 1).

CRP was measured by ELISA using a commercial kit (Abcam, UK) in the Swedish samples and by an immunoturbidimetric assay (Roche, CH) in the Portuguese samples.

All samples were blind-coded for the diagnoses.

### Statistical Analysis

#### Variables

*Outcome variable*: the diagnostic status, as categorical variables: SCC, MCI, AD and (MCI + AD). SCC was considered the reference group in the analysis, except when stated otherwise. Comparisons were also made between the MCI and AD groups whenever appropriate.

*Exposure variable:* LCN2 concentration, as continuous variable in serum (ng/ml) and CSF (pg/ml).

*Confounding variables*: Age in years, as a continuous variable, as it is the most important risk factor for MCI and AD (Gauthier et al., 2006; Winblad et al., 2016), and because it may also relate to LCN2 concentrations given the underlying change in the inflammatory status with aging (Mocchegiani et al., 2014). Number of years of formal education, as a continuous variable, since lower education is associated with both MCI and AD and because a higher educational level has been related to better health condition and habits impacting the individual’s inflammatory status (Ngandu et al., 2007; Meng and D’Arcy, 2012; Winblad et al., 2016). Finally, sex, because men might have a higher risk of MCI, women may have a higher risk for AD (especially among the older group), and because there may be sex-specific differences in risk profiles (Roberts et al., 2012; Prince et al., 2013; Mielke et al., 2014). Men were used as the reference category.

Variables were checked for normality using the Shapiro-Wilk test. For normally distributed variables comparisons between diagnosis groups were made using *t*-tests or ANOVA with Tukey *post hoc* analysis. For variables that did not follow a normal distribution, both parametric and non-parametric (Mann–Whitney and Kruskal–Wallis) tests were used. All variables except CSF and serum LCN2 levels in the Portuguese sample displayed normal distributions.

#### Statistical Tests

Explanatory binary logistic regression models were built considering dichotomous outcomes: SCC vs. (MCI + AD), and SCC vs. AD. Explanatory multinomial logistic regression models were also built for the diagnosis outcomes: SCC vs. MCI or AD, and MCI vs. SCC or AD. The regression models were checked for multicollinearity through correlation analysis of each pair of independent variables by Pearson’s correlation coefficient.

*p* < 0.05 and 95% confidence intervals were considered for statistically significance.

## Results

Table 1 summarizes descriptive statistics and comparisons. No significant differences were found in LCN2 levels in serum across groups in the Swedish and Portuguese cohorts. When pooled together, MCI and AD groups of the Swedish cohort showed a higher LCN2 serum level compared with the SCC control group. In the Swedish sample, CSF LCN2 levels were also higher in MCI and AD groups compared with the SCC controls. However, controls were significantly younger and had more years of education than individuals from the MCI and AD groups. On the contrary, in the Portuguese sample, where groups were identical with respect to sex and number of years of education, no differences were found in serum or CSF levels between SCC and AD groups.

**Table 1:** Characterization of the Swedish and Portuguese cohorts.

	Swedish cohort				Portuguese cohort^&^	
	SCC^1^	MCI^1^	AD^1^	(MCI + AD)^2^	SCC	AD
Sex, % women	68	65	68	67	67	63
Age in years, mean ± SEM	55 ± 1.3^a^	64 ± 1.3^b^	67 ± 1.6^b^	65 ± 1.1^b^	60 ± 1.3	64 ± 1.5
Formal education in years, mean ± SEM	14 ± 0.5^a^	12 ± 0.5^b^	11 ± 0.5^b^	11 ± 0.4^b^	7 ± 1.0 (*n* = 12)	5 ± 0.6 (*n* = 23)
Serum LCN2 in ng/ml, mean ± SEM*	89 ± 4.1^a^ (*n* = 33)	108 ± 6.2 (*n* = 34)	104 ± 6.2 (*n* = 38)	106 ± 4.4^b^ (*n* = 72)	151 ± 16.4 (*n* = 12)	178 ± 17.0 (*n* = 24)
CSF LCN2 in pg/ml, mean ± SEM*	769 ± 29.4^a^ (*n* = 34)	958 ± 44.7^b^ (*n* = 29)	957 ± 46.7^b^ (*n* = 30)	958 ± 32.1^b^ (*n* = 59)	1007 ± 71.5 (*n* = 12)	1226 ± 76.6 (*n* = 23)
Complete sample per group^#^	38	37	38	75	12	24

^1^Comparisons among SCC, MCI and AD were done using ANOVA. ^2^Student *t*-test was used to compare SCC vs. MCI + AD. Different letters (a,b) indicate statistically significant differences at *p* < 0.05. ^&^No comparisons were made between cohorts. *The LCN2 values in the Portuguese CSF and serum groups did not follow a normal distribution. SCC—subjective cognitive complaints; SEM—standard error of the mean. ^#^The reasons for the varied n in each group are provided in Figures 1;  2.

Next, we performed a binary logistic regression analysis for the outcome cognitive impairment (belonging to the MCI + AD group) considering as independent variables the LCN2 concentration in the serum and CSF, alone or together. Table 2 summarizes the findings for the Swedish cohort when the (MCI + AD) group is compared with the SCC group. In the crude analysis, there was 0.4% higher odds to be cognitively impaired (MCI + AD) for each pg/ml increase in the CSF LCN2 concentration. Similarly, there was 1.5% higher odds to be cognitively impaired (MCI + AD) for each ng/ml increase in the serum LCN2 concentration. When the LCN2 concentration in the two biological fluids was considered together, the association between LCN2 concentration and cognitive impairment (MCI + AD) remained only for the CSF levels. These associations were no longer significant in the adjusted model, after controlling for sex, education, and age.

**Table 2:** Crude and adjusted OR with 95% CI from binary logistic regression for the probability of having cognitive impairment (AD + MCI) using SCC as reference group for the Swedish groups.

		Crude		Adjusted*	
LCN2 levels	Risk group	OR (95% CI)	*P*-value	OR (95% CI)	*P*-value
CSF, pg/ml	(MCI + AD)	1.004 (1.001–1.006)	0.002	1.002 (0.999–1.005)	0.220
Serum, ng/ml	(MCI + AD)	1.015 (1.002–1.028)	0.026	1.013 (0.997–1.030)	0.109
CSF, pg/ml	(MCI + AD)	1.033 (1.001–1.006)	0.015	1.001 (0.998–1.005)	0.358
Serum, ng/ml		1.012 (0.994–1.030)	0.200	1.013 (0.992–1.035)	0.215

*Adjusted for sex, age in years, and formal education in years. AD—Alzheimer’s disease; CI—confidence intervals; CSF—cerebrospinal fluid; LCN2—lipocalin 2; MCI—mild cognitive impairment; OR—odds ratio; SCC—subjective cognitive complaints.

Table 3 shows the multinomial logistic regression analysis for the outcomes AD and MCI for the Swedish cohort. When compared to the SCC group, LCN2 concentration in the CSF (Table 3) was associated with both MCI and AD, with a 0.4% increase in the odds to have the disease for each pg/ml in CSF LCN2. Serum LCN2 concentration was only associated with MCI. The association of CSF and serum LCN2 concentration with MCI remained, but not that with AD, when both variables were considered together. All associations lost significance when adjusted for sex, education, and age.

**Table 3:** Crude and adjusted OR with 95% CI from multinomial logistic regression for the probability of having cognitive impairment (AD or MCI) using SCC as reference group for the Swedish groups.

		Crude		Adjusted*	
LCN2 levels	Risk group	OR (95% CI)	*P*-value	OR (95% CI)	*P*-value
CSF, pg/ml	MCI	1.004 (1.001–1.006)	0.004	1.002 (0.999–1.005)	0.160
	AD	1.004 (1.001–1.006)	0.004	1.001 (0.998–1.005)	0.414
Serum, ng/ml	MCI	1.016 (1.1001–1.031)	0.033	1.016 (0.999–1.034)	0.066
	AD	1.013 (0.999–1.027)	0.076	1.010 (0.992–1.028)	0.299
CSF, pg/ml	MCI	1.033 (1.004–1.006)	0.026	1.002 (0.998–1.005)	0.310
	AD	1.010 (0.990–1.030)	0.317	1.013 (0.991–1.036)	0.245
Serum, ng/ml	MCI	1.003 (0.001–1.006)	0.020	1.001 (0.998–1.005)	0.511
	AD	1.012 (0.993–1.032)	0.227	1.014 (0.990–1.038)	0.257

*Adjusted for sex, age in years, and formal education in years. AD—Alzheimer’s disease; CI—confidence intervals; CSF—cerebrospinal fluid; LCN2—lipocalin 2; MCI—mild cognitive impairment; OR—odds ratio; SCC—subjective cognitive complaints.

Regarding the Portuguese sample, no associations were found between LCN2 concentration, in the serum or CSF, alone or together, and AD, when compared to the reference control group (Table 4).

**Table 4:** Crude and adjusted OR with 95% CI from binary logistic regression for the probability of having cognitive impairment (AD) using SCC as reference group for the Portuguese groups.

		Crude		Adjusted*	
LCN2 levels	Risk group	OR (95% CI)	*P*-value	OR (95% CI)	*P*-value
CSF, pg/ml	AD	1.003 (1.000–1.006)	0.082	1.003 (0.999–1.006)	0.108
Serum, ng/ml	AD	1.005 (0.994–1.017)	0.343	1.004 (0.992–1.016)	0.531
CSF, pg/ml	AD	1.003 (0.996–1.006)	0.116	1.003 (0.999–1.006)	0.132
Serum, ng/ml		1.001 (0.987–1.014)	0.911	1.001 (0.986–1.016)	0.944

*Adjusted for sex, age in years, and formal education in years. AD—Alzheimer’s disease; CI—confidence intervals; CSF—cerebrospinal fluid; LCN2—lipocalin 2; MCI—mild cognitive impairment; OR—odds ratio; SCC—subjective cognitive complaints.

## Discussion

This study, using two independent cohorts, provides no clear evidence for the association of LCN2 concentrations in CSF and in serum with MCI or AD, after controlling for age, sex, and number of years of education. This observation is of relevance and must be interpreted together with other reports where LCN2 levels were associated with cognitive impairment (Choi et al., 2011; Naudé et al., 2012). None of these previous studies controlled for education, even though there were differences in the number of years of formal education among the groups (Choi et al., 2011). With respect to age, the individuals in the study by Naudé et al. (2012) were older than those in the present study and age-matched in the studied groups (controls, MCI, AD); Choi and collaborators (Choi et al., 2011) controlled for age but not for education, which was significantly lower in the AD group (when compared to MCI and to control). It would be interesting to confirm whether the differences in those two studies would remain after controlling for education, and also whether the association found in the present study would persist in an elderly population. A recent study in four cohorts, controlled for sex and age, found no differences in CSF LCN2 levels between controls and AD patients (Llorens et al., 2020).

Altogether, from the reported studies, it is clear that attention should be given to confounding variables when searching for an association of markers of disease. In the case of cognitive impairment, age is a key variable to consider, since the older the individuals the higher the risk to develop MCI and AD (Winblad et al., 2016). Education is protective for the development of cognitive impairment, and several studies have described that a higher number of years of formal education are associated with a lower risk to develop MCI and AD (Ngandu et al., 2007; Meng and D’Arcy, 2012). While the evidence is strong in that both age and education relate to the outcome, the evidence of their association with the independent variable LCN2 is weaker but must be considered. For any biological marker that may indicate a state of inflammation, as is the case of LCN2, it is conceivable that higher formal education may relate with information on health habits more likely to protect individuals from causes leading to inflammation; for that reason, education may relate with the levels of LCN2. As for age, independently from the common practice to control for age in most association studies, age is again a likely confounder when LCN2 is considered. Age is associated with the “aging” of several organ systems, including the immune system. It is currently accepted that several neurodegenerative disorders, and aging in itself, are associated with an underlying pro-inflammatory state, of which LCN2 could be a marker or be related to. In agreement, LCN2 levels have been shown to be positively correlated with age (De la Chesnaye et al., 2016; Maurizi et al., 2021). Furthermore, LCN2 levels have been described to be associated with several neurological neurodegenerative conditions (Marques et al., 2012; Maurizi et al., 2021; Ferreira et al., 2015), possibly as a general marker of inflammation rather than specifically associated with the disease. Interestingly, Llorens et al. (2020) reported that CSF LCN2 may specifically distinguish vascular dementia from Lewy body dementia, fronto-temporal dementia, Creutzfeldt-jakob disease, AD, and mixed dementia (AD plus vascular dementia). Additional studies are warranted to further investigate the cause of increased CSF LCN2 in such conditions, given the potential disruption of the blood-brain barrier. Altogether, these studies support the importance of confounding factors, like age and education, when searching for potential disease biomarkers, such as LCN2.

Attention should also be given to the diagnosis criteria. Eruysal et al. (2019) reported higher LCN2 plasma levels in individuals classified as preclinical AD in accordance with the CSF composition criteria.

The main limitation of the present study pertains to the cross-sectional design. In addition, even though the sample size exceeds the one calculated considering the expected effect size for group differences reported for LCN2 values in blood and CSF, it would be relevant to repeat this study in a larger cohort. Importantly, higher sample sizes and wider age groups might also be needed for any biomarkers displaying subtle changes. Additional prospective longitudinal studies and the inclusion of biomarkers of disease and disease progression will provide information on the potential usefulness of LCN2 in MCI/AD evaluation. Additionally, the storage duration of the samples, before LCN2 quantification, differs in the two cohorts, which could possibly influence LCN2 levels. Nevertheless, a previous study has demonstrated that serum LCN2 concentration presents a high stability along time (up to 7 days at 4°C) and after repetitive freeze/thaw cycles (up to three cycles; Wang et al., 2015). Moreover, no direct comparison was performed between the two cohorts, only between MCI/AD groups and the respective reference groups within each cohort, whose samples have been stored for similar time periods.

The strengths of the study reside on the use of two independent cohorts with detailed cognitive evaluation, the measurement of LCN2 in two biological fluids and the careful consideration for potential confounding factors.

Of note, while the present study strongly suggests that measuring LCN2 levels in biological fluids is not useful for diagnosis purposes, LCN2 may still be associated with the molecular mechanisms underlying cognitive impairment and/or AD. In accordance, studies in animals showed that in the absence of LCN2 rodents display: (i) anxious and depressive-like behaviors, as well as cognitive impairment in spatial learning tasks (Ferreira et al., 2013); (ii) deficits in adult neural stem cells proliferation and commitment, with impact on the hippocampal-dependent contextual fear discriminative task (Ferreira et al., 2018, 2019). In addition, *in vitro* studies suggest that LCN2 mediates the toxicity of Aβ protein to astrocytes (Mesquita et al., 2014).

In summary, the present study does not support LCN2 levels in serum and CSF as a useful marker in identifying individuals clinically characterized to have MCI or AD.

## Data Availability Statement

The raw data supporting the conclusions of this article will be made available by the authors, without undue reservation.

## Ethics Statement

The studies involving human participants were reviewed and approved by the Ethical Committee in Stockholm (ID number: 2007/697-31/1) and was approved by the GEDOC steering group and by the Ethics Committee at Centro Hospitalar Universitário do Porto (CHUP; ID number: 2011/1987-31/4). Written informed consent was obtained by all participants or legal representatives in the case of individuals with severe cognitive impairment unable to decide. All data were anonymized, managed, and analyzed according to international ethical standards. The patients/participants provided their written informed consent to participate in this study.

## Author Contributions

SPN and JAP performed the experiments. SPN, FM, and JAP performed the data analysis and wrote the manuscript. RT provided the Portuguese cohort samples. PS revised statistical analysis. JM-E and MK critically revised the manuscript. JAP designed and supervised the study and edited the manuscript. All authors contributed to the article and approved the submitted version.

## Conflict of Interest

The authors declare that the research was conducted in the absence of any commercial or financial relationships that could be construed as a potential conflict of interest.

## References

[B1] American Psychiatric Association (2005). Diagnostic and Statistical Manual of Mental Disorders: DSM-IV [Internet], 4th edition. Washington, DC: American Psychiatric Association. Available online at: http://www.psychiatryonline.com/DSMPDF/dsm-iv.pdf.

[B2] Almkvist O. Tallberg I. -M. (2009). Cognitive decline from estimated premorbid status predicts neurodegeneration in Alzheimer’s disease. Neuropsychology 23, 117–124. 10.1037/a0014074 19210039

[B3] Bi F. Huang C. Tong J. Qiu G. Huang B. Wu Q.. (2013). Reactive astrocytes secrete lcn2 to promote neuron death. Proc. Natl. Acad. Sci. U S A 110, 4069–4074. 10.1073/pnas.1218497110 23431168 PMC3593910

[B4] Choi J. Lee H. -W. Suk K. (2011). Increased plasma levels of lipocalin 2 in mild cognitive impairment. J. Neurol. Sci. 305, 28–33. 10.1016/j.jns.2011.03.023 21463871

[B5] De la Chesnaye E. Manuel-Apolinar L. Oviedo-de Anda N. Revilla-Monsalve M. C. Islas-Andrade S. (2016). Gender differences in lipocalin 2 plasmatic levels are correlated with age and the triglyceride/high-density lipoprotein ratio in healthy individuals. Gac. Med. Mex. 152, 612–617. 27792695

[B6] Eruysal E. Ravdin L. Kamel H. Iadecola C. Ishii M. (2019). Plasma lipocalin-2 levels in the preclinical stage of Alzheimer’s disease. Alzheimers Dement. 11, 646–653. 10.1016/j.dadm.2019.07.004 31517027 PMC6733778

[B7] Ferreira A. C. Mesquita S. D. Sousa J. C. Correia-Neves M. Sousa N. Palha J. A.. (2015). From the periphery to the brain: lipocalin-2, a friend or foe. Prog. Neurobiol. 131, 120–136. 10.1016/j.pneurobio.2015.06.005 26159707

[B8] Ferreira A. C. Novais A. Sousa N. Sousa J. C. Marques F. (2019). Voluntary running rescues the defective hippocampal neurogenesis and behavior observed in lipocalin 2-null mice. Sci. Rep. 9:1649. 10.1038/s41598-018-38140-y 30733506 PMC6367505

[B9] Ferreira A. C. Pinto V. Mesquita S. D. Novais A. Sousa J. C. Correia-Neves M.. (2013). Lipocalin-2 is involved in emotional behaviors and cognitive function. Front. Cell Neurosci. 7:122. 10.3389/fncel.2013.00122 23908604 PMC3725407

[B10] Ferreira A. C. Santos T. Sampaio-Marques B. Novais A. Mesquita S. D. Ludovico P.. (2018). Lipocalin-2 regulates adult neurogenesis and contextual discriminative behaviors. Mol. Psychiatry 23, 1031–1039. 10.1038/mp.2017.95 28485407

[B11] Gauthier S. Reisberg B. Zaudig M. Petersen R. C. Ritchie K. Broich K.. (2006). Mild cognitive impairment. Lancet 367, 1262–1270. 10.1016/S0140-6736(06)68542-5 16631882

[B12] Harada C. N. Love M. C. N. Triebel K. L. (2013). Normal cognitive aging. Clin. Geriatr. Med. 29, 737–752. 10.1016/j.cger.2013.07.002 24094294 PMC4015335

[B14] Katz M. J. Lipton R. B. Hall C. B. Zimmerman M. E. Sanders A. E. Verghese J.. (2012). Age-specific and sex-specific prevalence and incidence of mild cognitive impairment, dementia and Alzheimer dementia in blacks and whites: a report from the einstein aging study. Alzheimer Dis. Assoc. Disord. 26, 335–343. 10.1097/WAD.0b013e31823dbcfc 22156756 PMC3334445

[B15] Kulmala J. Ngandu T. Havulinna S. Levälahti E. Lehtisalo J. Solomon A.. (2019). The effect of multidomain lifestyle intervention on daily functioning in older people. J. Am. Geriatr. Soc. 67, 1138–1144. 10.1111/jgs.15837 30809801

[B16] León-Salas B. Ayala A. Blaya-Nováková V. Avila-Villanueva M. Rodríguez-Blázquez C. Rojo-Pérez F.. (2015). Quality of life across three groups of older adults differing in cognitive status and place of residence. Geriatr. Gerontol Int. 15, 627–635. 10.1111/ggi.12325 25109790

[B17] Lee S. Kim J.-H. Kim J.-H. Seo J.-W. Han H.-S. Lee W.-H.. (2011). Lipocalin-2 Is a chemokine inducer in the central nervous system: role of chemokine ligand 10 (CXCL10) in lipocalin-2-induced cell migration. J. Biol. Chem. 286, 43855–43870. 10.1074/jbc.M111.299248 22030398 PMC3243551

[B18] Lee S. Lee W.-H. Lee M.-S. Mori K. Suk K. (2012). Regulation by lipocalin-2 of neuronal cell death, migration and morphology. J. Neurosci. Res. 90, 540–550. 10.1002/jnr.22779 22038922

[B19] Lee S. Park J.-Y. Lee W.-H. Kim H. Park H.-C. Mori K.. (2009). Lipocalin-2 is an autocrine mediator of reactive astrocytosis. J. Neurosci. 29, 234–249. 10.1523/JNEUROSCI.5273-08.2009 19129400 PMC6664907

[B20] Llorens F. Hermann P. Villar-Piqué A. Diaz-Lucena D. Nägga K. Hansson O.. (2020). Cerebrospinal fluid lipocalin 2 as a novel biomarker for the differential diagnosis of vascular dementia. Nat. Commun. 11:619. 10.1038/s41467-020-14373-2 32001681 PMC6992814

[B21] Marques F. Mesquita S. D. Sousa J. C. Coppola G. Gao F. Geschwind D. H.. (2012). Lipocalin 2 is present in the EAE brain and is modulated by natalizumab. Front. Cell Neurosci. 6:33. 10.3389/fncel.2012.00033 22907989 PMC3414908

[B22] Maurizi A. Ponzetti M. Gautvik K. M. Reppe S. Teti A. Rucci N. (2021). Lipocalin 2 serum levels correlate with age and bone turnover biomarkers in healthy subjects but not in postmenopausal osteoporotic women. Bone Rep. 14:101059. 10.1016/j.bonr.2021.101059 34026950 PMC8121999

[B23] McKhann G. M. Knopman D. S. Chertkow H. Hyman B. T. Jr C. R. J. Kawas C. H.. (2011). The diagnosis of dementia due to Alzheimer’s disease: recommendations from the national institute on aging-Alzheimer’s association workgroups on diagnostic guidelines for Alzheimer’s disease. Alzheimers Dement. 7, 263–269. 10.1016/j.jalz.2011.03.005 21514250 PMC3312024

[B24] Meng X. D’Arcy C. (2012). Education and dementia in the context of the cognitive reserve hypothesis: a systematic review with meta-analyses and qualitative analyses. PLoS One 7:e38268. 10.1371/journal.pone.0038268 22675535 PMC3366926

[B25] Mesquita S. D. Ferreira A. C. Falcao A. M. Sousa J. C. Oliveira T. G. Correia-Neves M.. (2014). Lipocalin 2 modulates the cellular response to amyloid beta. Cell Death Differ. 21, 1588–1599. 10.1038/cdd.2014.68 24853299 PMC4158684

[B26] Mesquita S. D. Ferreira A. C. Sousa J. C. Santos N. C. Correia-Neves M. Sousa N.. (2012). Modulation of iron metabolism in aging and in Alzheimer’s disease: relevance of the choroid plexus. Front. Cell Neurosci. 6:25. 10.3389/fncel.2012.00025 22661928 PMC3357636

[B27] Mielke M. M. Vemuri P. Rocca W. A. (2014). Clinical epidemiology of Alzheimer’s disease: assessing sex and gender differences. Clin. Epidemiol. 6, 37–48. 10.2147/CLEP.S37929 24470773 PMC3891487

[B28] Mocchegiani E. Costarelli L. Giacconi R. Malavolta M. Basso A. Piacenza F.. (2014). Micronutrient-gene interactions related to inflammatory/immune response and antioxidant activity in ageing and inflammation. a systematic review. Mech. Ageing Dev. 136, 29–49. 10.1016/j.mad.2013.12.007 24388876

[B29] Mucha M. Skrzypiec A. E. Schiavon E. Attwood B. K. Kucerova E. Pawlak R. (2011). Lipocalin-2 controls neuronal excitability and anxiety by regulating dendritic spine formation and maturation. Proc. Natl. Acad. Sci. U S A 108, 18436–18441. 10.1073/pnas.1107936108 21969573 PMC3215032

[B30] Naudé P. J. W. Nyakas C. Eiden L. E. Ait-Ali D. Heide R. Engelborghs S.. (2012). Lipocalin 2: novel component of proinflammatory signaling in Alzheimer’s disease. FASEB J. 26, 2811–2823. 10.1096/fj.11-202457 22441986 PMC3382095

[B31] Ngandu T. Strauss E. V. Helkala E.-L. Winblad B. Nissinen A. Tuomilehto J.. (2007). Education and dementia: what lies behind the association. Neurology 69, 1442–1450. 10.1212/01.wnl.0000277456.29440.16 17909157

[B33] Petersen R. C. (2004). Mild cognitive impairment as a diagnostic entity. J. Intern. Med. 256, 183–194. 10.1111/j.1365-2796.2004.01388.x 15324362

[B34] Petersen R. C. Caracciolo B. Brayne C. Gauthier S. Jelic V. Fratiglioni L. (2014). Mild cognitive impairment: a concept in evolution. J. Intern. Med. 275, 214–228. 10.1111/joim.12190 24605806 PMC3967548

[B35] Pilling L. C. Harries L. W. Powell J. Llewellyn D. J. Ferrucci L. Melzer D. (2012). Genomics and successful aging: grounds for renewed optimism. J. Gerontol A Biol. Sci. Med. Sci. 67, 511–519. 10.1093/gerona/gls091 22454374 PMC3326244

[B36] Prince M. Bryce R. Albanese E. Wimo A. Ribeiro W. Ferri C. P. (2013). The global prevalence of dementia: a systematic review and metaanalysis. Alzheimers Dement. 9, e2.63–e2.75. 10.1016/j.jalz.2012.11.007 23305823

[B37] Prince M. Wimo A. Guerchet M. Ali G.-C. Wu Y.-T. Prina M. (2015). World Alzheimer Report 2015. The Global Impact of Dementia—An Analysis of Prevalence, Incidence, Costs and Trends. London: Alzheimer’s Disease International.

[B38] Rabin L. A. Smart C. M. Amariglio R. E. (2017). Subjective cognitive decline in preclinical Alzheimer’s disease. Annu. Rev. Clin. Psychol. 13, 369–396. 10.1146/annurev-clinpsy-032816-045136 28482688

[B39] Rathore K. I. Berard J. L. Redensek A. Chierzi S. Lopez-Vales R. Santos M.. (2011). Lipocalin 2 plays an immunomodulatory role and has detrimental effects after spinal cord injury. J. Neurosci. 31, 13412–13419. 10.1523/JNEUROSCI.0116-11.2011 21940434 PMC6623298

[B40] Roberts R. O. Geda Y. E. Knopman D. S. Cha R. H. Pankratz V. S. Boeve B. F.. (2012). The incidence of MCI differs by subtype and is higher in men: the mayo clinic study of aging. Neurology 78, 342–351. 10.1212/WNL.0b013e3182452862 22282647 PMC3280046

[B41] Roudkenar M. H. Halabian R. Bahmani P. Roushandeh A. M. Kuwahara Y. Fukumoto M. (2011). Neutrophil gelatinase-associated lipocalin: a new antioxidant that exerts its cytoprotective effect independent on heme oxygenase-1. Free Radic. Res. 45, 810–819. 10.3109/10715762.2011.581279 21545264

[B42] US Food and Drug Administration (2005). Review Criteria for Assessment of C-Reactive Protein (CRP), High Sensitivity C-Reactive Protein (hsCRP) and Cardiac C-Reactive Assays. Availabe online at: https://www.fda.gov/regulatory-information/search-fda-guidance-documents/review-criteria-assessment-c-reactive-protein-crp-high-sensitivity-c-reactive-protein-hscrp-and-0.

[B43] Wang J. Zhu H. H. Xue J. H. Wu S. S. Chen Z. (2015). Effects of storage conditions on the stability of serum CD163, NGAL, HMGB1 and MIP2. Int. J. Clin. Exp. Pathol. 8, 4099–4105. 26097598 PMC4466985

[B44] Winblad B. Amouyel P. Andrieu S. Ballard C. Brayne C. Brodaty H.. (2016). Defeating Alzheimer’s disease and other dementias: a priority for european science and society. Lancet Neurol. 15, 455–532. 10.1016/S1474-4422(16)00062-4 26987701

[B13] World Health Organization. (2011). Global Health and Aging. Available online at: https://www.who.int/ageing/publications/global_health.pdf. Accessed January 16, 2021.

[B32] World Health Organization. (2020). *Dementia*. Available online at: https://www.who.int/news-room/fact-sheets/detail/dementia.

